# Towards an Automated Approach to the Semi-Quantification of [^18^F]F-DOPA PET in Pediatric-Type Diffuse Gliomas

**DOI:** 10.3390/jcm12082765

**Published:** 2023-04-07

**Authors:** Enrico Peira, Francesco Sensi, Luca Rei, Ruben Gianeri, Domenico Tortora, Francesco Fiz, Arnoldo Piccardo, Gianluca Bottoni, Giovanni Morana, Andrea Chincarini

**Affiliations:** 1 Istituto Nazionale di Fisica Nucleare (INFN), 16146 Genoa, Italy; 2 Neuroradiology Unit, IRCCS Istituto Giannina Gaslini, 16147 Genoa, Italy; 3 S.C. di Medicina Nucleare, E.O. Ospedali Galliera, 16128 Genoa, Italy; 4 Department of Neurosciences, University of Turin, 10124 Turin, Italy

**Keywords:** semi-quantification, *SUVr*, [^18^F]F-DOPA PET, pediatric oncology, PDGs

## Abstract

Background: This study aims to evaluate the use of a computer-aided, semi-quantification approach to [^18^F]F-DOPA positron emission tomography (PET) in pediatric-type diffuse gliomas (PDGs) to calculate the tumor-to-background ratio. Methods: A total of 18 pediatric patients with PDGs underwent magnetic resonance imaging and [^18^F]F-DOPA PET, which were analyzed using both manual and automated procedures. The former provided a tumor-to-normal-tissue ratio (*T_N_*) and tumor-to-striatal-tissue ratio (*T_S_*), while the latter provided analogous scores (*t_n_*, *t_s_*). We tested the correlation, consistency, and ability to stratify grading and survival between these methods. Results: High Pearson correlation coefficients resulted between the ratios calculated with the two approaches: ρ = 0.93 (*p* < 10^−4^) and ρ = 0.814 (*p* < 10^−4^). The analysis of the residuals suggested that t_n_ and t_s_ were more consistent than *T_N_* and *T_S_*. Similarly to *T_N_* and *T_S_*, the automatically computed scores showed significant differences between low- and high-grade gliomas (*p* ≤ 10^−4^, *t*-test) and the overall survival was significantly shorter in patients with higher values when compared to those with lower ones (*p* < 10^−3^, log-rank test). Conclusions: This study suggested that the proposed computer-aided approach could yield similar results to the manual procedure in terms of diagnostic and prognostic information.

## 1. Introduction

Pediatric-type diffuse gliomas (PDGs) comprise a heterogeneous group of central nervous system tumors, ranging from low-grade to highly malignant tumors, characterized by different biological behaviors and molecular profiles when compared with their adult counterparts [1].

Among the different diagnostic techniques, magnetic resonance imaging (MRI) represents the current imaging modality of choice in the evaluation of pediatric gliomas not only because it is safe due to the absence of radiation exposure but also for its spatial and contrast resolution [2]. Beyond conventional MRI, advanced MRI techniques including perfusion weighted imaging (PWI), diffusion tensor imaging (DTI), and MR spectroscopy (MRS) can also add hemodynamic, microstructural, and metabolic information under a multiparametric approach. Indeed, multiparametric MRI has been demonstrated to provide valuable information for tumor characterization and grading, treatment planning, and post-treatment surveillance [3,4].

In parallel with the increased application of multiparametric MRI, a growing body of evidence suggests that positron emission tomography (PET) imaging with radiolabelled amino acids can provide further and complementary insights into pediatric glioma evaluation complementing MRI, as recently outlined in the Joint EANM/SIOPE/RAPNO practice guidelines/SNMMI procedure standards for imaging of pediatric gliomas using PET with radiolabelled amino acids and [^18^F]FDG [5]. Of note, to reduce concerns regarding the radiation dose associated with PET/CT, which represents a potential limitation of this technique, it is essential to optimize and harmonize the acquisition protocols of pediatric PET/CT to use reasonably low radiation doses while maintaining acceptable image quality and duration [6]. Among amino acid PET tracers, [^18^F]F-DOPA has demonstrated a high potential in defining tumor grade and outcome in PDGs; furthermore, it can be used for biopsy planning and treatment surveillance (e.g., discrimination between disease progression and treatment-related changes) [7,8,9,10,11,12,13].

Visual evaluation of the PET scan by a trained nuclear medicine physician is often complemented by semi-quantitative measures that estimate the tracer uptake in PET scans. Standardized uptake value (*SUV*) represents the most used technique due to the simplicity of its application and its efficacy in assessing response to treatment [14]. Yet, many different factors can affect the *SUV*, for instance, the time interval between the acquisition and scanning, the image acquisition setting, and the algorithm used to define the tumor. Therefore, it is difficult to compare *SUVs* acquired in different centers when acquisition and analysis protocols are not shared and agreed upon [15,16]. Great efforts are currently being made to standardize the computation of the *SUV* to enable comparison across and between centers [17].

The most common ways to report SUVs are the *SUV_mean_* and the *SUV_max_*, which are the mean or maximum *SUV* of all voxels within a target region of interest (*ROI*). The ratio between two *SUVs*, calculated between a target *ROI* and a reference one, is named as the standardized uptake value ratio (*SUVr*). *ROIs* are often manually drawn on a single, bidimensional slice of the scan, which is a non-trivial, time-consuming task. The size and placement of *ROIs*, for instance, may be inadequate to establish the pathological and healthy tissue uptake references. Furthermore, the definition of the *ROIs* can make the procedure largely operator dependent and the use of a single bidimensional slice of the image, without exploiting the information coming from the volume of the lesion, can cause a loss of information. All these issues are likely to influence the semi-quantification, and their effect could potentially be mitigated by the introduction of automatic methods to support clinicians in the estimation of the *SUVr*.

Based on these considerations, the aim of this study was to develop a computer-aided, semi-quantification method on [^18^F]F-DOPA PET/CT images co-registered with MRIs of PDGs. The proposed method mimics the manual procedure described by Morana et al. [8].

## 2. Materials and Methods

Briefly, the study by Morana et al. [8] relies on the human assessment performed on a fused PET/MRI visualization. A square-shaped *ROI* (side 1.8 cm) was drawn manually on the axial section of the tumor area exhibiting the highest [^18^F]F-DOPA uptake. In cases where the increase in uptake was not apparent, a *ROI* of the same size was manually placed on the bulk of the lesion according to standard diagnostic criteria. The radiotracer concentration in the *ROI* was then normalized to the injected dose per patient’s body weight, and the *SUV_max_* was calculated for each lesion (*SUV_max,T_*; g/mL). A same-sized *ROI* was mirrored in the matching contralateral hemisphere, and another was drawn over the contralateral striatum, centered on the putamen. *SUV_max_* values were calculated for each *ROI* (*SUV_max,N_* and *SUV_max,S_*; g/mL). The lesion-to-normal-tissue uptake (*t_n_*) and lesion-to-striatal uptake (*t_s_*) ratios were calculated by dividing the *SUV_max,T_* by the *SUV_max,N_* and by the *SUV_max,S_*, respectively.

(1) $$\begin{array}{c} T_{N}=\frac{SUV_{max,T}}{SUV_{max,N}}\\ T_{S}=\frac{SUV_{max,T}}{SUV_{max,S}} \end{array}$$

We sought a semi-quantification algorithm which does not need to manually define uptake and reference *ROI*s of fixed size and shape; rather, we explored a more robust way to locate data-driven regions that represent the uptake of unaffected tissue, thus improving on the normalization values of the lesion. Similarly to the procedure described above, we looked for two normalization values representing the average uptake of the healthy tissue and striatum, respectively. These values were used to normalize the whole image, from which we extracted the two *SUVr* corresponding to the scores estimated with the manual procedure. In the development of the method, we were blind to the manual *SUVr* and to the clinical data (e.g.,: survival, tumor grading) that were used only retrospectively to conduct the validation analysis.

Some minimal assumptions were made in the definition of the automated approach. First, we assumed that gliomas were characterized by an increased uptake of [^18^F]F-DOPA [18]. We also assumed that the approximate volume of each tumor was significantly smaller than the brain parenchyma volume. Additionally, we assumed that the gliomas were predominantly monolateral. The latter hypothesis was visually verified on the analyzed dataset. Therefore, the regions that best represented the uptake of the healthy tissue were obtained starting from the contralateral, non-lesioned brain hemisphere.

### 2.1. Patients

Our dataset consisted of 18 patients: 7 females and 11 males aged from 5 to 17 (µ = 10.3 ± 3.9) who underwent PET study due to the presence of an infiltrating lesion on conventional MRI. The MRI and [^18^F]F-DOPA PET were acquired within a 2-week window. Overall, there were 9 pediatric-type diffuse low-grade gliomas (PLGG; all with WHO grade II [1,5]) and 9 pediatric-type diffuse high-grade gliomas (PHGG; 4 patients with WHO grade III and 5 with WHO grade IV [1,5]). Patients’ demographics and lesion characteristics are summarized in Table 1. 

### 2.2. Image Protocol

[^18^F]F-DOPA was purchased from a commercial supplier (IASOdopa; IASON Labormedizine Ges.Mbh & Co., KG, Graz-Seiersberg, Austria). Volumetric scans were acquired 20 min after the injection of 185 MBq of a median injected activity of [^18^F]F-DOPA, with a dedicated PET/CT system (Discovery ST; GE Healthcare, Milwaukee, WI, USA) using the three-dimensional mode with a scanning time of 30 min. A non-diagnostic low-dose CT scan (120 kV, 80 mA, 0.6 s per rotation) was used for attenuation correction. The interpretation of all the [^18^F]F-DOPA PET scans involved a qualitative analysis based on visual assessment, as well as a semi-quantitative analysis using the *SUVr*.

MRI studies were performed on a 1.5 T magnet (Intera Achieva, Philips, Best, The Netherlands). The routine brain MRI examination consisted of axial fluid attenuation inversion recovery (FLAIR) and T1-weighted (T1w) images (axial, coronal and sagittal) acquired after gadolinium chelate bolus administration (0.1 mmol/kg).

### 2.3. Spatial Normalization

The target PET image was registered to the corresponding MRI T1w with a 7-parameter transformation and the mutual information metric. An automatic multi-step registration procedure involving three subsequent steps of increasing accuracy (linear registration with 6, 7, and 12 degrees of freedom) was applied to map the T1w onto the MNI space (isotropic spacing and voxel dimension of 1 × 1 × 1 mm, see Figure S1; Supplementary Materials). The resulting transformation was applied to the corresponding PET image which was thereby mapped to MNI coordinates. Hereinafter, we refer to the PET mapped on MNI coordinates as P and each voxel of P will be denoted as *p* (*p* ∈ P).

Once all the MR images (e.g., T1w and FLAIR) and the PET scans were registered onto the MNI space, we proceeded with a multi-step, post-processing stage to find the uptake values that could be representative of healthy, striatum, and tumorous tissue. The spatial normalization pipeline was implemented with the Insight Toolkit (ITK, version 4.12).

### 2.4. Identification of Brain Non-Affected Hemisphere

The following steps were all implemented in Matlab (version R2017b). To discriminate between injured and healthy tissue, we trained a support vector machine (SVM) over all the FLAIR images of the dataset. Indeed, FLAIR has proven to be effective in tumor delineating even in PLGG [19]. Any single scan was independently parcelled into ~300 districts by means of an intensity-based K-means algorithm. Among all the generated districts (~18 × 300), a randomized sample of 360 regions was labelled as healthy or pathological by a trained neuroradiologist. We then selected 300 volumes to form a balanced set of affected and non-affected regions.

**Figure 1:**
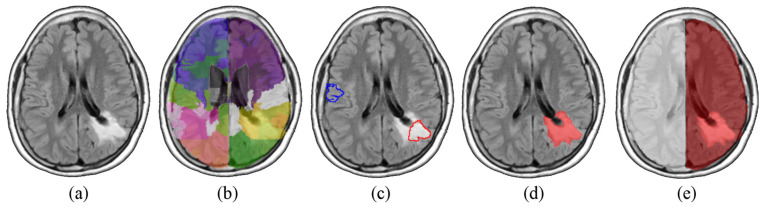
Steps (axial sections) of the procedure for the preliminary tumor mass localization. Each FLAIR image is mapped onto the MNI space (**a**), and (**b**) parcelled into ~300 districts (different colors identify different areas). In (**c**) clear training regions for SVM are selected (typical healthy and affected tissue outlined in blue and red respectively), and in (**d**) all the areas classified as diseased are merged together in a volume (V_t_, in red). Final decision of the tumor-affected hemisphere (right hemisphere, highlighted in red), by intersecting V_t_ with a coarse mask of the two hemispheres (**e**).

In analogy to what was accomplished by Soltaninejad et al. [20], the SVM was fed with a set of 14 texture features computed on each region (i.e., average, standard deviation, variance, mean and median of the absolute deviation, skewness, kurtosis, maximum, minimum, median and mode of the intensity values, range, interquartile range, and entropy). The classifier was then tested on the whole dataset giving out a three-dimensional mask (V_t_), for every image, containing the affected volume. The position of V_t_ allowed us to identify the hemisphere in which each lesion was predominantly located. All these steps are illustrated in Figure 1.

Notably, the SVM classification was not used to segment the tumor. Rather, it was only used as seed to define the affected and non-affected brain hemisphere. Therefore, segmentation accuracy is not a concern as any coarse segmentation would serve the purpose.

### 2.5. Reference Volume Extraction

The contralateral hemisphere (assumed as mostly unaffected) was divided into 5 lobes (V_i_, i = 1, …, 5) by application of the MNI atlas, with the exclusion of the pons and the striatum. Since all V_i_ belong to the non-affected hemisphere, we expected that they could be used to estimate the general intensity distribution of the whole unaffected tissue (reference). From each V_i_ we computed a reference tissue region M_i_ (i = 1, …, 5): we applied a moving-window filter that compares the intensity statistics in a neighborhood I(*p*) surrounding each voxel *p* of the brain, with that of V_i_. For simplicity we chose a cubic shaped sliding window centered in *p* (I(*p*) = (2l + 1)^3^ with l = 3 voxels). To efficiently describe the intensity distribution of V_i_, we sampled its histogram into K = 10 quantile bins. The set of voxels whose intensity distribution in I(*p*) is not different from V_i_, are assigned to M_i_. This comparison has been accomplished by applying a threshold to an χ^2^-like function *Y* (Equation (2)). The threshold on *Y* was heuristically set to 10^−3^.

(2) $$Y=\sum_{j=1}^{k=10}\frac{{\left(o_{j}-e_{j}\right)}^{2}}{e_{j}}$$

where *e_j_* is the reference intensity distribution (the one from V_i_, intensity quantile *j*) and *o_j_* is the observed distribution from the sliding window I(*p*).

**Figure 2:**
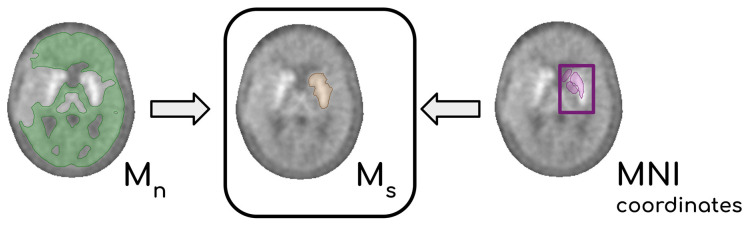
Diagram showing the processing that leads to the definition of M_s_. By integrating the information on the position of the basal ganglia (retrieved from the normalization to the MNI coordinates, purple area within the purple box) with the mask M_n_ obtained with the *Y*-filter (green region), it is possible to delineate a volume M_s_ that contains the whole striatum (yellow area).

The final mask M_n_ was then computed by averaging the five M_i_. In the end, each PET P was provided with a mask M_n_ containing the normal, reference, tissue. An infographic of the workflow, for the extraction of M_n_, is summarized in Figure S2a (Supplementary Materials). Each mask M_n_ was usually a large, connected area consisting of non-affected tissue. Its intensity characteristics showed a mono-modal distribution, which is coherent with the removal of districts showing significantly different uptake (Supplementary Materials, Figure S2b). The arithmetic mean c_n_ of the intensities in M_n_ was chosen to be the representative of the normal tissue. Finally, the normalized image P_n_ was obtained dividing the intensity of each voxel of P by c_n_. An analogous procedure was carried out to normalize P to the activity of the normal striatum. Briefly, the uptake of the striatum in [^18^F]F-DOPA scans allowed the *Y*-filter to operate a coarse segmentation of subcortical structures. Knowing the position of the striata from the spatial normalization to MNI coordinates, we could outline a volume M_s_ containing points discarded from *Y* which in turn contained the whole striatum located in the non-affected hemisphere. From M_s_ a representative value of the striatal activity c_s_ was extracted. In Figure 2, the steps leading to the definition of M_s_ are summarized. Target PET P voxels were then divided by c_s_, and the resulting P_s_ is the PET scannormalized to the mean activity of the normal striatum.

### 2.6. Maximum SUVr Extraction

**Figure 3:**
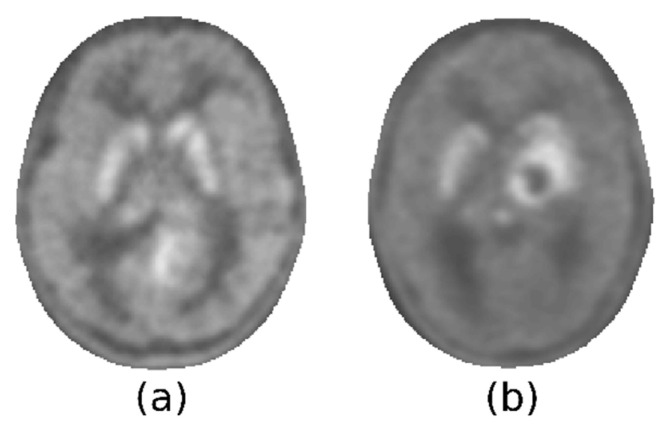
Axial section showing a tumor that does not affect striatum (**a**) and one that has reached it (**b**).

The maximum intensities within the lesions, in P_n_ and P_s_, are conceptually analogous to the *T_N_* and *T_S_* derived with the manual procedure (Equation (1)). If we denote by M_c_ all the voxels of the brain except M_n_ and M_s_, the mask obtained contains both the striatum within the lesioned hemisphere and the glioma. To compare the results of the two procedures, it was necessary to identify a value corresponding to the local maximum intensity of the lesion, and this search was limited to M_c_. There are two criticalities in the definition of this value. The first is related to the presence of the striatum in the volume M_c_. This subcortical structure presents a tracer uptake that could be comparable to that of the glioma. Consequently, it is possible for a minimum of two hyperintense areas to be observable, one of which may remain unaltered by the tumor and where the highest degree of uptake may be localized. The key point here is that it was not possible to discriminate the increased uptake of the healthy striatum from the increased uptake of lesions. It was also impossible, relying only on [^18^F]F-DOPA PET, to determine whether the infiltration of the striata by the glioma had clearly occurred. Figure 3 illustrates two examples of this heterogeneity: the tumor could present with less, more, or as much activity as the striatum, which could be spared by the tumor or not.

This consideration led us to abandon the idea of providing a single maximum within the lesion from P_n_ and P_s_. Therefore, the adopted solution was to provide two maximum values: one located within the striatum (m_in_) and one located outside of it (m_out_). Two scenarios are possible: if m_out_ ≥ m_in_, then m_out_ is selected to represent the maximum activity of the glioma, otherwise the involvement of the basal ganglia by the tumor must be evaluated. When m_out_ < m_in_, two options arise: (1) infiltration of glioma in the basal ganglia is evident, and therefore m_in_ can be considered a reliable marker of tumor activity, or (2) if the infiltration is unclear, it is reasonable to regard m_out_ as the most representative value, which is frequently observed in low-grade gliomas. To find m_in_, it was necessary to identify a binary mask (M_c,s_) containing the striatum inside M_c_. This operation is conceptually similar to the one performed to obtain M_s_, but in this case, the raw segmentation operated by the *Y*-filter was not sufficient. Indeed, we needed to consider that extended gliomas can deform surrounding tissues, with subcortical structures included. Thus, we chose to create M_c,s_ by inflating the raw mask obtained through the filter to be entirely encircling the striatum located in the affected hemisphere. The expansion of M_c,s_ was influenced by the intensity of neighboring voxels, implying that M_c,s_ could potentially incorporate affected tissue. The fraction of M_c_ not containing M_c,s_ represents affected tissue, except for the striatum in the impacted hemisphere; we refer to this volume as M_c,n_ (M_c_ = M_c,s_ ∪ M_c,n_). The most appropriate value between m_in_ and m_out_ was then chosen by expert clinicians taking into consideration their coordinates and the extension of each glioma.

**Figure 4:**
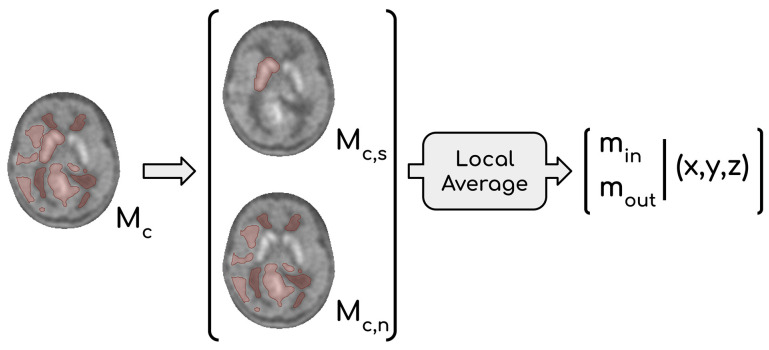
Extraction of the two local maxima: m_in_ and m_out_. M_c_ is the mask containing all the voxels rejected by the *Y*-filter. From this mask, we can derive two masks M_c,n_ and M_c,s_ so that M_c_ = M_c,s_∪M_c,n_. M_c,s_ is obtained by inflating the raw mask obtained through the *Y*-filter to be entirely encircling the striatum. M_c,n_ is calculated as the difference M_c_ − M_c,s_. The two local maxima m_in_ and m_out_ are the highest among the local averages calculated in M_c,s_ and M_c,n_, respectively. This operation is carried out using a cubic shaped window centered in each *p* (in this case l = 2). Finally, a set of coordinates is reported for both m_in_ and m_out_; these coordinates refer to the center of the cubic windows whose mean intensity is the highest.

The second issue concerned the choice of a single voxel value as maximum intensity; indeed, the intensity of just one voxel is prone to fluctuation and noise. Thus, it was decided to calculate local averages using a moving window approach and to consider them as representative of tumor malignancy. The size of the cubic moving window was set to be comparable to those of the *ROI*s used in the manual procedure (l = 2). Values m_out_ and m_in_ were consequently the highest local means computed in M_c,n_ and M_c,s_, respectively. Let m_1_, …, m_n_ be the mean intensities in the local neighborhood I(*p*_1_), …, I(*p*_n_) with *p*_1_, …, *p*_n_ ∈ M_c,s_, then m_in_ = max{m_1_, …, m_n_ }. Similarly, m_out_ = max{m_1_, …, m_n_ } where *p*_1_, …, *p*_n_ ∈ M_c,n_. The whole procedure that leads to the definition of both m_in_ and m_out_ is summarized in Figure 4.

The selection of the local mean was substantiated by the consistent observation that the examined tumors demonstrated a maximum uptake in the central region, which gradually declined towards the periphery.

This entire procedure was performed on both P_n_ and P_s_, providing two scores that reflected the maximum activity in the diseased tissue normalized, respectively, by the mean activity in the healthy tissue (*t_n_*) and by the mean activity of the striatum (*t_s_*).

### 2.7. Evaluation Metrics

The correlation between automated and manual results has been quantified by the Pearson correlation coefficient ρ between the scores obtained with the manual (*T_N_*, *T_S_*) and the automatic (*t_n_*, *t_s_*) procedure. We also verified the internal coherence of each semi-quantification procedure by comparing the residual dispersion of the linear regression of *t_s_*∼*t_n_* with that of the model *T_S_*∼*T_N_*.

An unpaired *t*-test was used to test the scores with respect to the lesion grading for the purpose of evaluating the potential diagnostic relevance.

The potential prognostic value of *t_n_* and *t_s_* was assessed by performing a survival analysis with the Kaplan–Meier (KM) method that estimates the survival function. A cut-off value *t_n_*_,cut_ for *t_n_* scores was calculated to maximize the sensitivity in the classification of prognosis: *t_n_*_,cut_ is defined as the value that minimizes the rate of false negatives and all subjects with *t_n_* < *t_n_*_,cut_ survived. To assess the discriminatory capability of the *t_n_*_,cut_ in stratifying patients with distinct prognoses, we compared the KM curves of two subsets based on their *t_n_*. The same procedure (cut-off calculation and KM curve analysis) was repeated with the *t_s_* values.

## 3. Results

**Figure 5:**
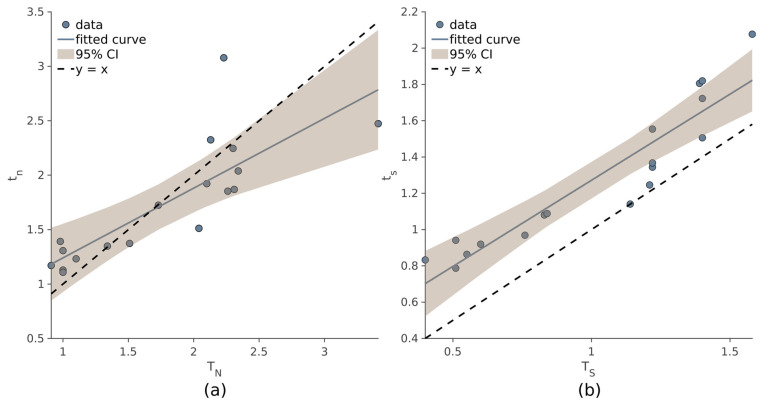
Relationship between *T_N_* and *t_n_* (**a**), and between *T_S_* and *t_s_* (**b**). In each scatter plot, the subjects are denoted with dots, the blue line is the linear model, and the brown area represents the 95% confidence interval. The dashed line is the bisector y = x.

As evidenced by the correlation coefficients, the values *t_n_* and *t_s_* are comparable to those obtained with the manual procedure (Equation (1)). Strong correlation was observed between *T_N_* and *t_n_* (ρ = 0.814, *p* < 10^−4^), and even higher values of ρ were observed between *T_S_* and *t_s_* (ρ = 0.93, *p* < 10^−4^). In Figure 5, the linear regression models of *t_n_*~*T_N_* (a) and *t_s_*~*T_S_* (b) are displayed.

**Figure 6:**
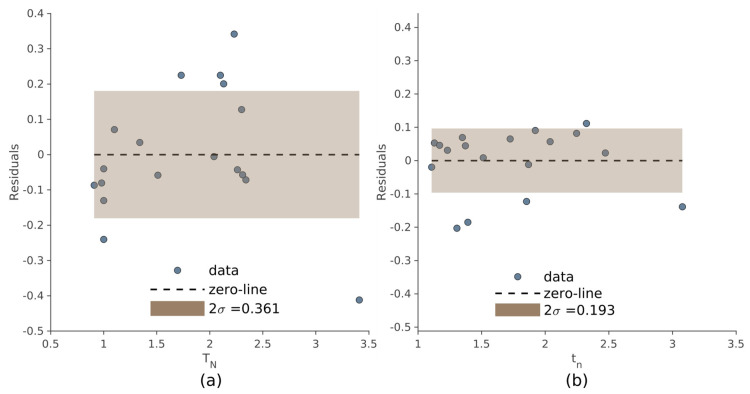
Residuals’ amplitude of the linear regressions *t_s_*∼*t_n_* (**a**) and *T_S_*∼*T_N_* (**b**). The brown area represents the standard deviation of the residuals: 2σ = 0.193 in (**a**) and 2σ = 0.361 in (**b**).

The dispersion of the residuals, used to evaluate the internal consistency of the two methods, is shown in Figure 6 referring to the manual and automated approach. Compared to the manual method, the automated procedure produced less dispersion of the residuals (σ = 0.181 and σ = 0.096, respectively).

**Figure 7:**
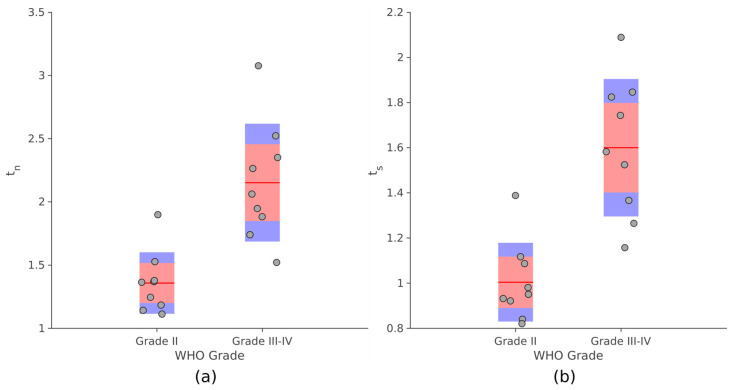
Distributions of *t_n_* (**a**) and *t_s_* (**b**) grouped by histological degree. Dots (subjects) are shown with the mean (red line), the 95% confidence interval on the median (pink band) and the interquartile range (blue band).

As Figure 7 displays, significant differences were observed for both *t_n_* and *t_s_* between PLGG and PHGG (unpaired *t*-test, *p* ≤ 10^−4^). Analogous results were obtained comparing the values provided by the manual procedure with the degree of malignancy (Supplementary Material, Figure S3).

**Figure 8:**
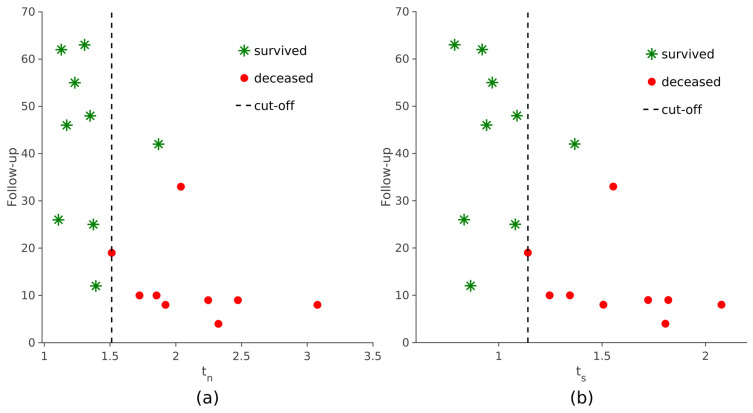
Follow-up (months) on the y-axis and semi-quantification scores on the x-axis. The patients are grouped by prognosis: green asterisks represent those who survived and red dots are those who did not survive. The dashed lines correspond to the two cut-offs: *t_n_*_,cut_ = 1.512 (**a**) and *t_s_*_,cut_ = 1.141 (**b**).

**Figure 9:**
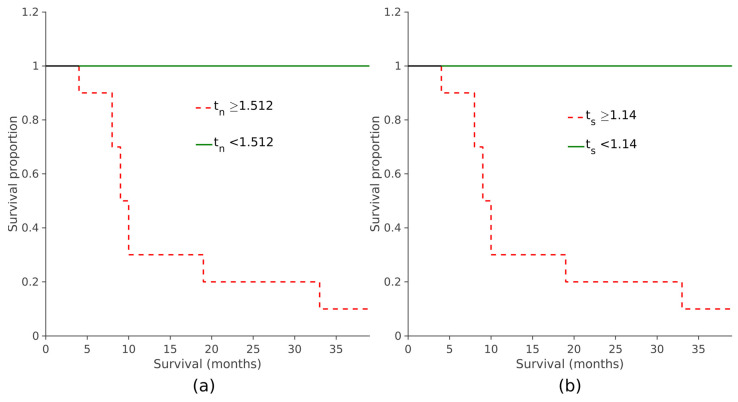
Survival curves discriminated by *t_n_*_,cut_ = 1.512 (**a**) and by *t_s_*_,cut_ = 1.141 (**b**). The red dashed curves represent the patients with *t_n_* ≥ *t_n_*_,cut_ (**a**) and *t_s_* ≥ *t_s_*_,cut_ (**b**). The steps indicate the instances at which the adverse events occurred; on the y-axis the probability of survival at a specific time interval is represented on the x-axis. The solid green curve (patients with *t_n_* (**a**) or *t_s_* (**b**) below the respective cut-off) remains flat during the whole observation period because there are no patients for which the adverse events took place.

When assessing the prognostic value of the two automatically derived scores, it was observed that elevated *t_n_* and *t_s_* values were linked to reduced overall survival, indicating poorer prognoses (*p* < 0.001, log-rank test; see Figure 8 and Figure 9). The same survival analysis was replicated with the maximum *SUVr* scores obtained with the manual procedure. Subjects were divided based on their scores and a KM curve was estimated for each group (Supplementary Materials, Figure S4). It was also observed in this case that the overall survival was significantly shorter in patients with higher values of *T_N_* and *T_S_* (*p* < 0.001, log-rank test).

## 4. Discussion

Approaches for analyzing [^18^F]F-DOPA PET/CT images, either semi-automated or fully automated, have been developed for use in Parkinson’s disease, where they can benefit from prior knowledge of the target and reference regions. A variety of techniques can be employed, including the extraction of radiomic features from the image, the application of classical machine learning methods, and the calculation of the striatal-to-occipital ratio [21,22]. Defining target and normalization regions in oncological PET imaging is a challenging task due to the inherent variability and the methodological drawbacks of common manual or threshold-based procedures (e.g., time consumption, low reproducibility, scanner type, reconstruction algorithm, and image noise [23]). This also certainly applies to the quantitative approach to [^18^F]F-DOPA PET [24,25,26]. However, as far as we know, there is currently no method for extracting unbiased quantitative information from neuro-oncological [^18^F]F-DOPA, whereas methods for such extraction are available for adults [^18^F]FET PET analysis [27,28]. Furthermore, a more in-depth analysis of the effect of reference region selection to improve analysis robustness has been conducted only on [^18^F]FET PET reporting that the selection of small bidimensional or three-dimensional *ROI*s is associated to significant background SUV changes that may be attributed to the potential differences in size, the inadequate representation of various tissue types, as well as the imprecise and arbitrary placement [29]. To the best of our knowledge, this is the only approach that allows for a significant reduction in intra- and inter-reader variability in defining the reference region on [^18^F]FET PET of adults with glioblastoma multiforme. This method, developed by Brighi and colleagues, involves the generation of a mirrored *ROI* of the lesion in the contralateral hemisphere with respect to the anterior–posterior midline [28]. Nonetheless, this approach may not be appropriate for patients who have tumor lesions significantly affecting regions located along the anterior–posterior midline, patients whose tumor growth has substantially compromised anatomical symmetry in the contralateral lobe, or those with multifocal bilateral diseases.

The method proposed in the present study mimics the manual routine adopted in clinical practice [8]. This automated approach could be useful in the definition of the *ROIs* drawn on tumor, striata, and normal brain parenchyma, in a [^18^F]F-DOPA PET scan, thus speeding up computation and reducing operator dependence for lesion-to-normal-tissue and lesion-to-striatal uptake ratios (*T_N_* and *T_S_*, respectively).

To validate this approach, we first verified the correlation of *t_n_* and *t_s_* with the *SUVr* computed with the manual procedure (*T_N_* and *T_S_*, respectively). An excellent correlation exists between the *T_S_* and *t_s_*, while a weaker, but still high, correlation was reached between *T_N_* and *t_n_*. The residual dispersion of the linear regression of *T_S_* and *T_N_* is greater than the same dispersion computed for *t_s_* and *t_n_*, suggesting that the scores issued from the automated procedure are more consistent than the couple of manually generated values. The consistency measured by the residual analysis assumes that normal tissue and normal striatum are in close relationship. That is to say that both are proxies of a tumor-free region, and hence, they can be thought of as the same measure. Moreover, the difference between the *SUVr* values is less evident between the *T_S_* and *t_s_* in determining a simple shift towards lower values for manual scores. This finding may be explained by the positioning of the reference *ROI* on the striatum body in the manual procedure. Indeed, the fixed-sized *ROI*s used to compute the T_S_ have always been placed in the center of the putamen where the uptake of the [^18^F]F-DOPA is higher than other cerebral tissues increasing the stability of these values [30]. Thus, we imply that (even though *SUV_max_* is less sensitive to *ROI* definition) the 1.8 × 1.8 cm squared *ROI* used in the manual procedure to obtain the *T_N_* can be more prone to uncertainty than the respective normal tissue reference c_n_ obtained from the extended volumes M_n_. Moreover, our data highlighted the ability of the *t_n_* and *t_s_* scores to distinguish between PLGGs and PHGGs in this study population. These results confirm the literature findings on [^18^F]F-DOPA uptake to discriminate between high- and low-degree lesions [7,8,9,10,11].

The survival analysis showed significant differences in patient prognosis for the subjects characterized by high *t_n_*, *t_s_* with respect to those having lower values.

The same analysis, performed on *T_N_* and *T_S_*, yielded comparable results suggesting that, for this population, a cut-off for discriminating between patients with significant differences in prognosis could be derived from both the automated and manual scores.

### Study Limitations

The inadequacy of the automatic procedure to assess striatal involvement of gliomas, which is beyond the purposes of the method itself, led to the decision of providing two local maxima for the *SUVr* estimation. However, a study conducted on pediatric patients with PDGs suggested that [^18^F]F-DOPA PET does not appear to be a main limitation in the evaluation of tumors extending into the basal ganglia but fused [^18^F]F-DOPA PET/MRI can be used to assess the striatal involvement [31]. Thus, MRI/PET co-registered scans coupled with the positions of two local maxima are adequate to allow a correct identification of the most appropriate site for *SUVr* estimation. The proposed method was tested on a small sample, and needs to be evaluated on a greater, multicentric dataset. However, our cohort included only patients with supratentorial lesions presenting an infiltrative pattern on MRI who underwent [^18^F]F-DOPA PET, which is rather uncommon, especially in the pediatric population. The automatic procedure was implemented without regard to tumor segmentation, rather, we were only interested in obtaining sufficiently large, connected volumes to ensure a stable representation of brain healthy tissue uptake. Thus, all the parameters of the filter (i.e., the size of the sliding window, the number K of quantiles used to sample the histogram) were estimated according to this principle, although their range could be further optimized.

## 5. Conclusions

This study suggests that computer-aided, semi-quantification approaches can yield similar results to the manual procedure in terms of diagnostic and prognostic information. The proposed method also proved to be more reliable as shown by internal cross checks. The results suggest that we can reduce the operator dependence and complement the visual inspection. Consequently, this approach may help to standardize the *SUVr* computation and enable the comparison among different centers in future prospective studies.

## 6. Compliance with Ethical Standards

The authors declare that they have no conflict of interest. All procedures performed in studies involving human participants were in accordance with the ethical standards of the institutional and/or national research committee and with the 1964 Helsinki declaration and its later amendments or comparable ethical standards. Written informed consent was signed by all participants or their legal guardians.

## Figures and Tables

**Table 1:** Patient characteristics, imaging findings and outcome. Abbreviations: PLLG, pediatric-type diffuse low-grade glioma; PHGG, pediatric-type diffuse high-grade glioma; *T_N_*, ratios of tumor-to-normal tissue uptake; *T_S_*, ratios of tumor-to-normal striatal uptake; DOD, died of disease; PD, progressive disease; SD, stable disease; PR, partial response; FU, follow-up in months.

Case	Age	Gender	Diagnosis	*T_N_*	*T_S_*	WHO Grade	Outcome	FU (Months)
1	12	F	PLGG	1.00	0.51	2	PR	63
2	10	M	PLGG	0.91	0.51	2	SD	46
3	14	M	PLGG	1.00	0.40	2	SD	26
4	11	F	PLGG	1.51	0.83	2	PR	25
5	5	M	PLGG	0.98	0.55	2	SD	12
6	9	M	PLGG	1.00	0.60	2	SD	62
7	8	M	PLGG	2.31	1.22	2	PD	42
8	13	M	PLGG	1.34	0.84	2	SD	48
9	15	M	PLGG	1.10	0.76	2	PD	55
10	6	F	PHGG	1.73	1.22	3	PD and DOD	10
11	8	F	PHGG	2.10	1.40	3	PD and DOD	8
12	14	M	PHGG	2.04	1.14	3	PD and DOD	19
13	16	F	PHGG	3.41	1.40	3	PD and DOD	9
14	6	F	PHGG	2.23	1.58	4	PD and DOD	8
15	8	M	PHGG	2.30	1.40	4	PD and DOD	9
16	5	M	PHGG	2.26	1.21	4	PD and DOD	10
17	8	M	PHGG	2.13	1.39	4	PD and DOD	4
18	17	F	PHGG	2.34	1.22	4	PD and DOD	33

## Data Availability

No new data were created or analyzed in this study. Data sharing is not applicable to this article.

## Supplementary Materials

The following supporting information can be downloaded at: https://www.mdpi.com/article/10.3390/jcm12082765/s1, Figure S1: The illustration summarizes the steps made to spatially register target MRI/PET images to the MNI reference space: PET image is registered to its correspondent T1w; MRI was mapped onto the MNI space (3 registration steps with 6, 7 and 12 degrees of freedom), and the subsequent PET to MNI mapping. Native orientations can be obtained using the inverse transforms T^−1^; Figure S2: The illustration (a) summarizes the steps towards the definition of the M_n_ mask. Each starting volume V_i_ provides an intensity distribution that is used as reference in filter *Y*. The filter compares the reference histogram with the intensity distribution contained in a cubic shaped moving window. This operation is conceptually analogous to a region growing and provides, for each V_i_, a three-dimensional mask M_i_ whose intensity distribution is similar to the distribution in V_i_. Finally, M_n_ is obtained by averaging the binarized masks M_i_ (i = 1, …, 5). In (b) an example of the mono-modal distribution of the intensities within M_n_ is displayed (green), and the gray distribution refers to the intensities within the whole brain. The c_n_ (red line) corresponds to the intensity average in M_n_; Figure S3: *T_N_* (a) and *T_S_* (b) values grouped by histological degree. Each dot represents a patient; the red line is the median, the pink band represents the 95% confidence level on the mean and the blue band is the interquartile range; Figure S4: Survival curves discriminated by *T_N_*_,cut_ = 1.73 (a) and by *T_S_*_,cut_ = 1.14 (b). The solid green curve (patients with *T_N_* (a) or *T_S_* (b) below the respective cut-off) remains flat during the whole observation period because there are no patients for which the adverse events took place. On the y-axis the probability of survival at a specific time-interval (x-axis).
