# Supplementary material for: Towards an Automated Approach to the Semi-Quantification of [18F]F-DOPA PET in Pediatric-Type Diffuse Gliomas
Source: J Clin Med. 2023 Apr 7;12(8):2765. doi: 10.3390/jcm12082765 (PMC10142802; doi:10.3390/jcm12082765)
Supplement: Supplementary file 1 [file jcm-12-02765-s001.zip › jcm-2165788-supplementary.pdf]

Towards an automated approach to the semi-quantification of [^{18}F]F-DOPA PET in pediatric-type diffuse gliomas

Supplementary material

[Authors]
Enrico Peira [1], Francesco Sensi [1], Luca Rei [1], Ruben Gianeri [1], Domenico Tortora [2], Francesco Fiz [3], Arnoldo Piccardo [3], Gianluca Bottoni [3], Giovanni Morana [4], Andrea Chincarini [1]

[Affiliations]
[1] Istituto Nazionale di Fisica Nucleare (INFN), 16146, Genoa, Italy;
[2] Neuroradiology Unit, IRCCS Istituto Giannina Gaslini, 16147, Genoa, Italy;
[3] S.C. di Medicina Nucleare, E.O. Ospedali Galliera, 16128, Genoa, Italy;
[4] Department of Neurosciences, University of Turin, 10124, Turin, Italy;

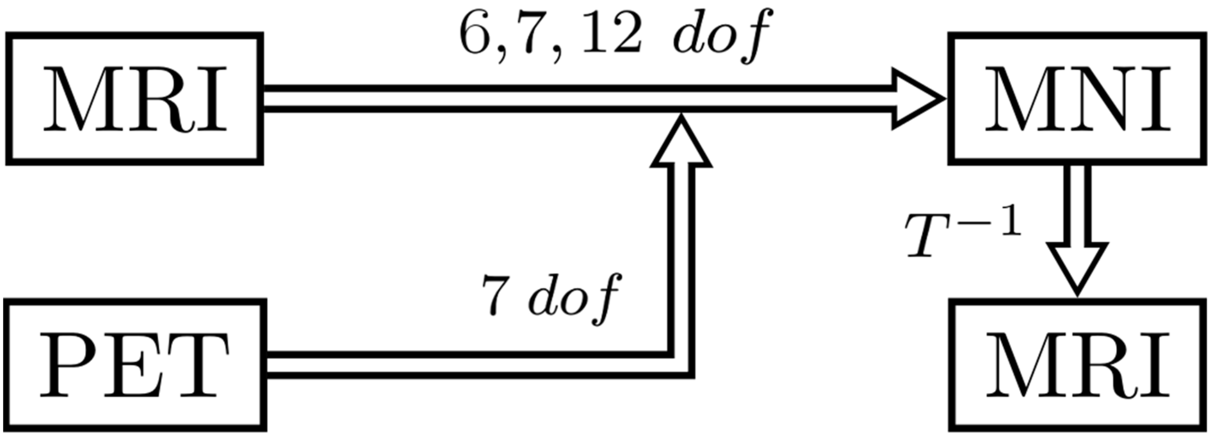

Figure S1 The illustration summarizes the steps made to spatially register target MRI/PET images to the MNI reference space: PET image is registered to its correspondent T1w; MRI was mapped onto the MNI space (3 registration steps with 6, 7 and 12 degrees of freedom), and the subsequent PET to MNI mapping. Native orientations can be obtained using the inverse transforms T^{-1}

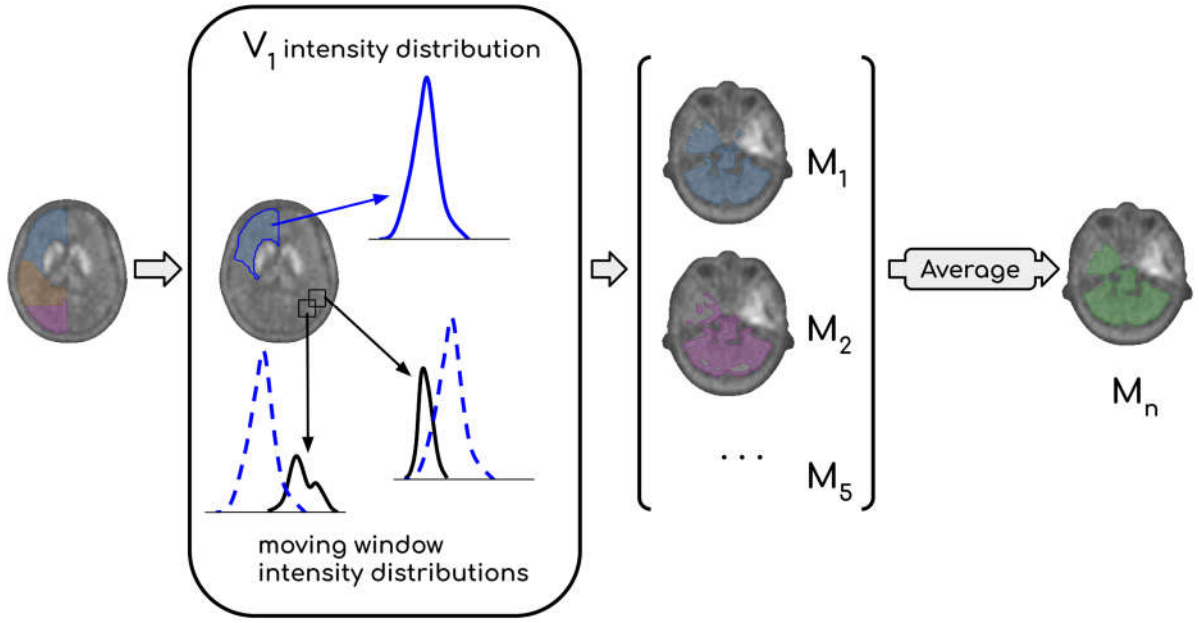

(a)

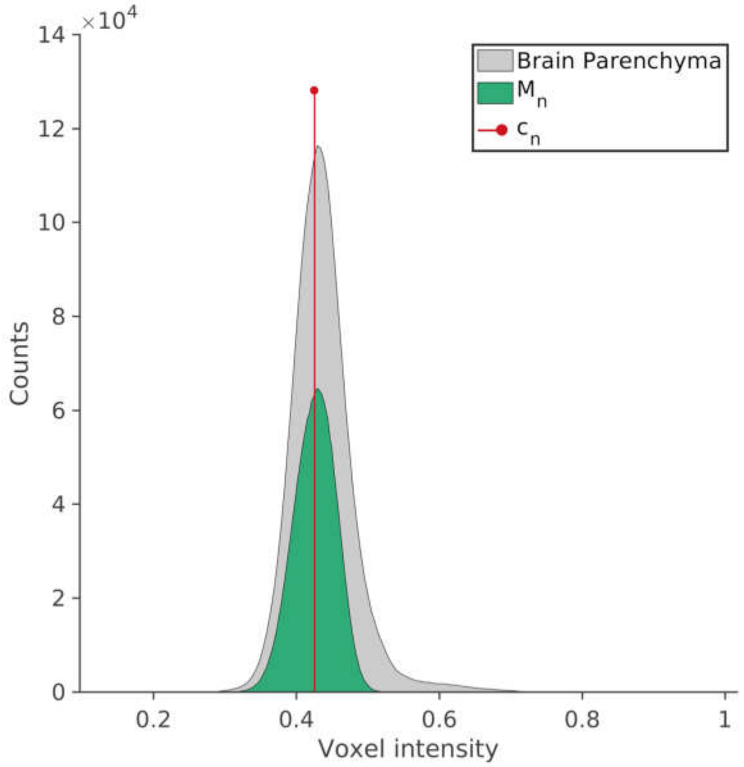

(b)

Figure S2 The illustration (a) summarizes the steps towards the definition of the M_n mask. Each starting volume V_i provides an intensity distribution that is used as reference in the filter Y. The filter compares the reference histogram with the intensity distribution contained in a cubic shaped mobile window. This operation is conceptually analogous to a region growing and provides, for each V_i, a three-dimensional mask M_i whose intensity distribution is similar to the distribution in V_i. Finally, M_n is obtained by averaging the binarized masks M_i (i=1, . . ., 5). In (b) an example of the mono-modal distribution of the intensities within M_n is displayed (green), and

the gray distribution is referred to the intensities within the whole brain. The c_n (red line) corresponds to the intensity average in M_n.

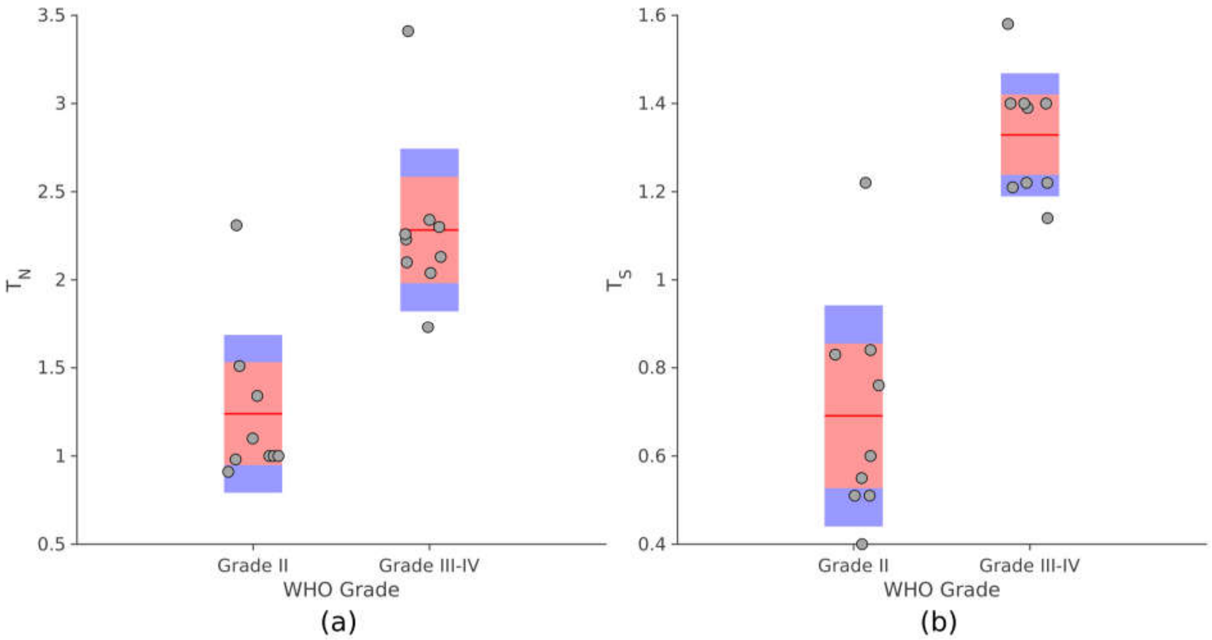

Figure S3 T_N (a) and T_S (b) values grouped by histological degree. Each dot represents a patient; the red line is the median, the pink band represents the 95% confidence level on the mean and the blue band is the interquartile range.

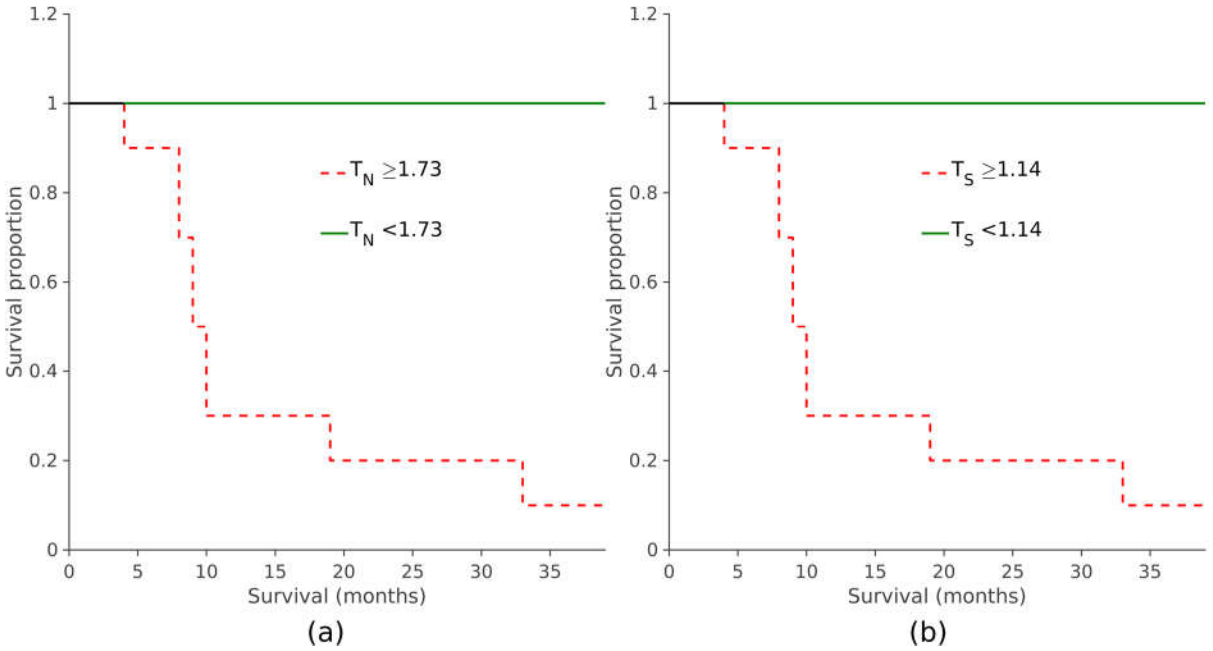

Figure S4 Survival curves discriminated by $T_{N,cut}$=1.73 (a) and by $T_{S,cut}$=1.14 (b).
The solid green curve (patients with T_N (a) or T_S (b) below the respective cut-off) remains flat during the whole observation period because there are no patients for which the adverse events took place. On the y-axis the probability of survival at a specific time-interval (x-a).
